# Adipocyte-specific deletion of *Tcf7l2* induces dysregulated lipid metabolism and impairs glucose tolerance in mice

**DOI:** 10.1007/s00125-020-05292-4

**Published:** 2020-10-17

**Authors:** Marie-Sophie Nguyen-Tu, Aida Martinez-Sanchez, Isabelle Leclerc, Guy A. Rutter, Gabriela da Silva Xavier

**Affiliations:** 1 Section of Cell Biology and Functional Genomics, Department of Metabolism, Digestion and Reproduction, Hammersmith Hospital, Imperial College Centre for Translational and Experimental Medicine, London, UK; 2 Lee Kong Chian School of Medicine, Nanyang Technological University, Singapore, Singapore; 3 Institute of Metabolism and Systems Research, University of Birmingham, Birmingham, UK

**Keywords:** Adipocyte, Beta cell, Fatty acid, Incretin, Insulin, Mouse, TCF7L2, Type 2 diabetes

## Abstract

**Aims/hypothesis:**

Transcription factor 7-like 2 (TCF7L2) is a downstream effector of the Wnt/β-catenin signalling pathway implicated in type 2 diabetes risk through genome-wide association studies. Although its expression is critical for adipocyte development, the potential roles of changes in adipose tissue TCF7L2 levels in diabetes risk are poorly defined. Here, we investigated whether forced changes in *Tcf7l2* expression in adipocytes affect whole body glucose or lipid metabolism and crosstalk between disease-relevant tissues.

**Methods:**

*Tcf7l2* was selectively ablated in mature adipocytes in C57BL/6J mice using *Cre* recombinase under *Adipoq* promoter control to recombine *Tcf7l2* alleles floxed at exon 1 (referred to as aTCF7L2 mice). aTCF7L2 mice were fed normal chow or a high-fat diet for 12 weeks. Glucose and insulin sensitivity, as well as beta cell function, were assessed in vivo and in vitro. Levels of circulating NEFA, selected hormones and adipokines were measured using standard assays.

**Results:**

Reduced TCF7L2 expression in adipocytes altered glucose tolerance and insulin secretion in male but not in female mice. Thus, on a normal chow diet, male heterozygote knockout mice (aTCF7L2het) exhibited impaired glucose tolerance at 16 weeks (*p* = 0.03) and increased fat mass (1.4 ± 0.1-fold, *p* = 0.007) but no changes in insulin secretion. In contrast, male homozygote knockout (aTCF7L2hom) mice displayed normal body weight but impaired oral glucose tolerance at 16 weeks (*p* = 0.0001). These changes were mechanistically associated with impaired in vitro glucose-stimulated insulin secretion (decreased 0.5 ± 0.1-fold vs control mice, *p* = 0.02) and decreased levels of the incretins glucagon-like peptide-1 and glucose-dependent insulinotropic polypeptide (0.6 ± 0.1-fold and 0.4 ± 0.1-fold vs control mice, *p* = 0.04 and *p* < 0.0001, respectively). Circulating levels of plasma NEFA and fatty acid binding protein 4 were increased by 1.3 ± 0.1-fold and 1.8 ± 0.3-fold vs control mice (*p* = 0.03 and *p* = 0.05, respectively). Following exposure to a high-fat diet for 12 weeks, male aTCF7L2hom mice exhibited reduced in vivo glucose-stimulated insulin secretion (0.5 ± 0.1-fold vs control mice, *p* = 0.02).

**Conclusions/interpretation:**

Loss of *Tcf7l2* gene expression selectively in adipocytes leads to a sexually dimorphic phenotype, with impairments not only in adipocytes, but also in pancreatic islet and enteroendocrine cells in male mice only. Our findings suggest novel roles for adipokines and incretins in the effects of diabetes-associated variants in *TCF7L2*, and further illuminate the roles of *TCF7L2* in glucose homeostasis and diabetes risk.

**Figure:**
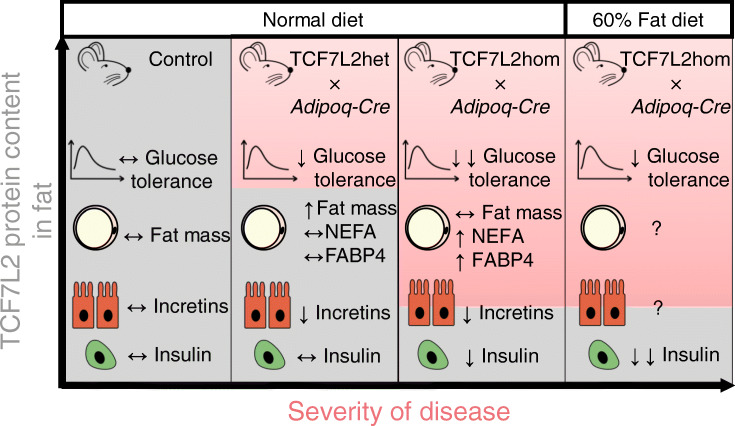
Graphical abstract

**Electronic supplementary material:**

The online version of this article (10.1007/s00125-020-05292-4) contains peer-reviewed but unedited supplementary material, which is available to authorised users.

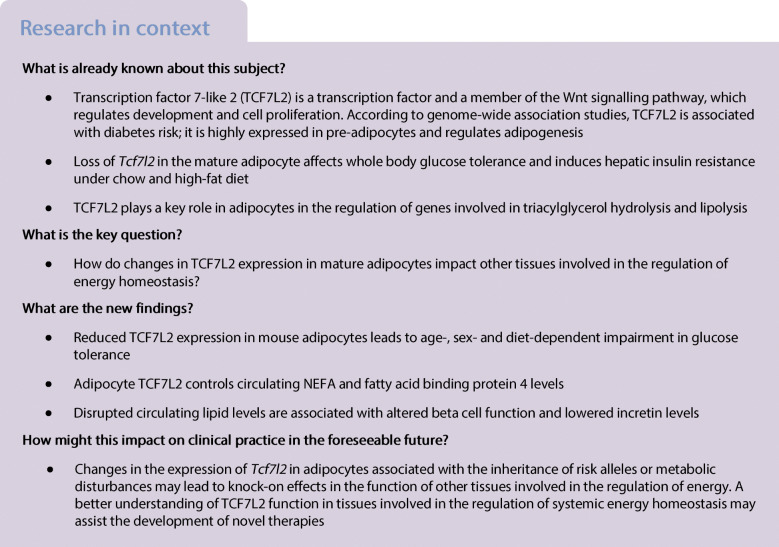



## Introduction

Transcription factor 7-like 2 (TCF7L2) is a member of the high mobility group box family of transcription factors, a downstream effector of the Wnt/β-catenin signalling pathway, and a key regulator of development and cell growth [1]. TCF7L2 has also emerged as an important regulator of energy homeostasis. Thus, TCF7L2 is required for normal glucose homeostasis via the maintenance of functional pancreatic beta cell mass and insulin release from the endocrine pancreas [2, 3]. On the other hand, the role(s) of this factor in other metabolically important tissues are less clear cut. For example, ablation of *Tcf7l2* expression from hepatocytes has variously been shown to lead to reduced hepatic glucose production and improved glucose homeostasis [4] or to hyperglycaemia [5].

The possible involvement of TCF7L2 in type 2 diabetes pathophysiology became apparent following the identification in genome-wide association studies of SNPs in the *TCF7L2* gene as amongst the most strongly associated with an increased risk of type 2 diabetes (reviewed in [6]). Humans carrying the T risk allele at SNP rs7903146 have elevated proinsulin levels, lowered insulin secretion and impaired responses to the incretin hormone glucagon-like peptide 1 (GLP-1) [7, 8]. While *TCF7L2* variants in humans are thought chiefly to act via pancreatic beta cell function, the mechanisms driving impaired insulin secretion are still poorly defined [9, 10]. Previous studies of the relationship between *TCF7L2* expression and glucose homeostasis have suggested that the combined effects of loss of *TCF7L2* in multiple tissues may underlie exaggerated diabetes risk [11].

The expression of *TCF7L2* has been shown to be reduced in adipose tissue from individuals with type 2 diabetes [12] and in obese mice [13], indicating that TCF7L2 function in adipose tissue may influence diabetes risk. MacDougald and colleagues [14, 15] have demonstrated that Wnt signalling is involved in regulating the expression of pro-adipogenic genes during adipocyte development. Recent studies have also implicated TCF7L2 as an important regulator of adipocyte differentiation and function [13, 16]. In addition, insulin and insulin growth factor 1 (IGF-1) have been shown to mediate crosstalk with the Wnt signalling pathway to regulate insulin sensitivity in pre-adipocytes [17]. The presence of TCF7L2 binding sites on the promoter of the insulin receptor gene also suggests a role for TCF7L2/Wnt signalling pathway in regulating insulin action in adipocytes [18]. However, the role of TCF7L2 in maintaining normal adipose tissue crosstalk in adult mice between adipocytes (important integrators of systemic energy homeostasis), and other tissues, is currently unclear.

In the present report, we have used *Tcf7l2* ablation selectively in mature adipocytes in mice to explore these questions. We have focused in particular on whether loss of *Tcf7l2* expression in the adipocyte may impact circulating levels of adipokines, incretins or insulin. In this way, we sought to explore the possibility that altered *TCF7L2* expression in the adipocyte may contribute to type 2 diabetes risk by affecting crosstalk between multiple tissues involved in energy homeostasis.

## Methods

Detailed descriptions of the experimental procedures can be found in the Electronic supplementary material (ESM).

### Animals

To achieve tissue-selective ablation of *Tcf7l2* alleles, we crossed mice in which exon 1 of *Tcf7l2* was flanked by *LoxP* sites [2] to mice expressing *Cre* recombinase under the control of the *Adipoq* promoter [19] to produce deletion of a single (aTCF7L2het) or two *Tcf7l2* alleles (aTCF7L2hom). Animals were housed in a pathogen-free facility with 12 h light–dark cycle with free access to a standard mouse chow (RM-1; Special Diet Services, UK) diet and water. High-fat diet (HFD) cohorts were placed on a high-sucrose, high-fat diet (D12331; Research Diets, New Brunswick, NJ, USA) for 12 weeks from 7 weeks of age. For the chow diet cohort, metabolic exploration was performed on each animal within a 2 week window at each stage (8-week-old and 16-week-old mice). All in vivo procedures described were performed at the Imperial College Central Biomedical Service and approved by the UK Home Office Animals Scientific Procedures Act, 1986 (PPL PA03F7F0F).

### In vivo metabolic assays

Glucose and insulin tolerance were assessed on fasted mice after oral or i.p. administration of glucose or insulin. Blood was collected to assess plasma insulin levels after oral or i.p. administration of glucose. Plasma insulin was measured using an ELISA kit (Crystal Chem, Netherlands) or a homogeneous time-resolved fluorescence (HTRF) kit (Cisbio, France).

### Protein isolation and Western immunoblotting

Antibodies used for immunoblotting are TCF4/TCF7L2 (C48H11) (#2569, 1:500, Cell signalling, NEB, UK), phospho-AKT (#9271, 1:1000, Cell signalling, NEB, UK), total-AKT (#9272, 1:1000, Cell signalling, NEB, UK), GAPDH (#2118, 1:10000, Cell signalling, NEB, UK) and alpha-tubulin (T5168, 1:10000, Sigma-Aldrich, UK).

#### Pancreatic islet isolation and in vitro insulin secretion

Pancreatic islets were isolated by collagenase digestion. Insulin secretion assays were performed on batches of ten size-matched islets and incubated for 30 min in Krebs-Ringer HEPES bicarbonate (KHB) solution with glucose (3–17 mmol/l) or KCl (30 mmol/l). Secreted and total insulin were quantified using a HTRF kit.

#### Intracellular free calcium imaging

Intact isolated islets were incubated with Fura-8 AM (Invitrogen, UK) and incubated in KHB containing glucose (3–17 mmol/l) or KCl (30 mmol/l). Ca^2+^-dependent fluorescence was imaged using a Nipkow spinning disk head (Yokogawa CSU-10; Runcorn, UK).

#### Analysis of circulating factors in plasma and serum

Blood was obtained from the tail vein of mice in the fed state. The concentrations of GLP-1, glucose-dependent insulinotropic polypeptide (GIP), leptin, adiponectin, plasminogen activator inhibitor-1 (PAI-1), fatty acid binding protein 4 (FABP4), resistin, NEFA and dipeptidyl peptidase 4 (DPP4) were measured using the kits detailed in ESM Methods.

### Histology

Epididymal adipose tissue was harvested, fixed overnight in 10% (*v*/v) formalin and embedded in paraffin wax. Tissue slices (5 μm) were stained with H&E (Sigma-Aldrich, UK) for morphological analysis using a widefield Axiovert 200M microscope (Zeiss, Germany) in the Facility for Imaging by Light Microscopy (Imperial College London).

### RNA isolation and quantitative PCR

RNA was isolated from epididymal and subcutaneous adipose tissue, liver and pancreatic islets. Gene expression was determined by quantitative RT-PCR. Primer sequences are listed in ESM Table 1.

### Statistical analysis

GraphPad Prism 8.4 was used for statistical analysis (GraphPad Software, USA). Significance was evaluated by unpaired Student *t* tests and one- or two-way ANOVA, with Tukey’s multiple comparison test. A *p* value of <0.05 was considered statistically significant. Data are shown as mean ± SEM.

## Results

### Reduced TCF7L2 expression in adipose tissue does not affect body weight but increases fat mass

**Fig. 1:**
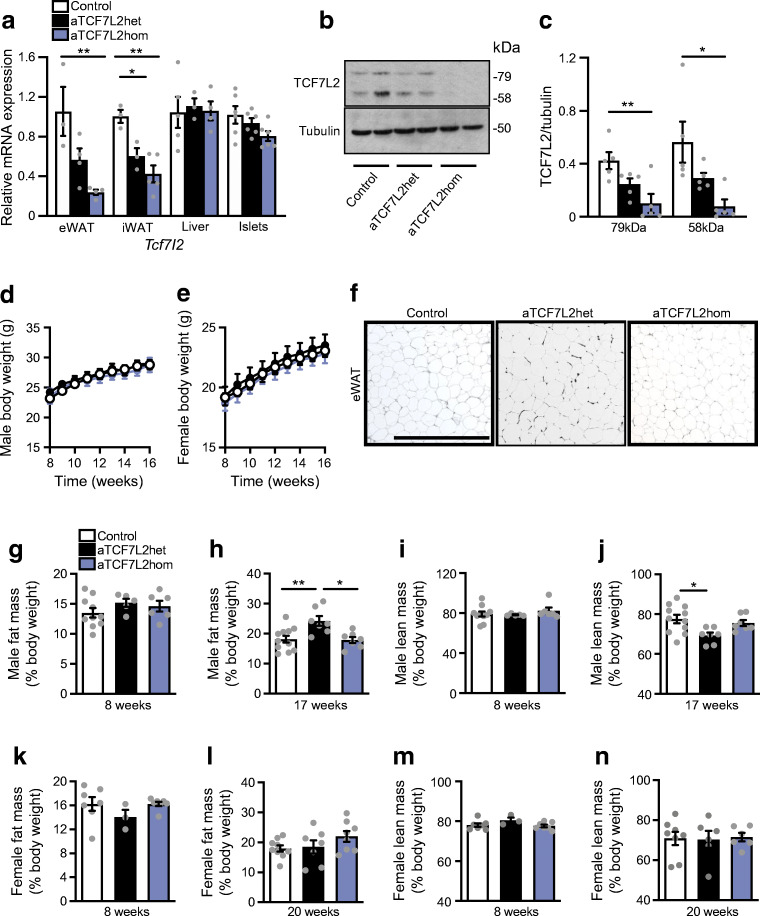
Effects of adipocyte-selective *Tcf7l2* deletion on body weight and fat mass in male and female mice on an NC diet. (**a**) *Tcf7l2* mRNA expression by quantitative RT-PCR in eWAT, iWAT, liver and isolated islets with *Gapdh*, *Actb* or *Ppia* used as internal normalisation control genes (*n* = 3–6 mice/genotype). One-way ANOVA with Tukey’s post hoc test, **p < 0.01 aTCF7L2hom and *p < 0.05 aTCF7L2het vs control, respectively. (**b**) Representative western blot of TCF7L2 protein expression in eWAT. (**c**) Densitometry quantification of TCF7L2 expression by western blotting in eWAT (*n* = 5 mice/genotype). One-way ANOVA with Tukey’s post hoc test, **p < 0.01 and *p < 0.05 aTCF7L2hom vs control. (**d**) Body weight in male and (**e**) female mice on an NC (males: *n* = 10 control mice, *n* = 9 aTCF7L2het mice, *n* = 6 aTCF7L2hom mice; females: *n* = 13 control mice, *n* = 7 aTCF7L2het mice, *n* = 7 aTCF7L2hom mice). (**f**) H&E staining of eWAT from mice on an NC. Scale bar, 100 μm. (**g**) Fat mass in 8-week-old males (*n* = 10 control mice, *n* = 5 aTCF7L2het mice, *n* = 6 aTCF7L2hom mice), (**h**) in 17-week-old males (*n* = 11 control mice, *n* = 6 aTCF7L2het mice, *n* = 7 aTCF7L2hom mice). One-way ANOVA with Tukey’s post hoc test, **p < 0.01 aTCF7L2het vs control and *p < 0.05 aTCF7L2hom vs aTCF7L2het. (**i**) Lean mass in 8-week-old males (*n* = 10 control mice, *n* = 5 aTCF7L2het mice, *n* = 6 aTCF7L2hom mice) and (**j**) in 17-week-old males (*n* = 11 control mice, *n* = 6 aTCF7L2het mice, *n* = 7 aTCF7L2hom mice). One-way ANOVA with Tukey’s post hoc test, *p < 0.05 aTCF7L2het vs control. (**k**) Fat mass in 8-week-old females (*n* = 7 control mice, *n* = 3 aTCF7L2het mice, *n* = 7 aTCF7L2hom mice) and (**l**) in 20-week-old females (*n* = 9 control mice, *n* = 7 aTCF7L2het mice, *n* = 8 aTCF7L2hom mice). (**m**) Lean mass in 8-week-old females (*n* = 7 control mice, *n* = 3 aTCF7L2het mice, *n* = 7 aTCF7L2hom mice) and (**n**) in 20-week-old females (*n* = 9 control mice, *n* = 7 aTCF7L2het mice, *n* = 8 aTCF7L2hom mice). Data shown as mean ± SEM

We generated a mouse line in which *Tcf7l2* was deleted selectively in adipocytes through the expression of *Cre* recombinase under the control of the *Adipoq* promoter. In aTCF7L2het mice, *Tcf7l2* mRNA levels were decreased in inguinal adipose tissue (iWAT) by 39.8 ± 13.3%, *p* = 0.02, while in epididymal white adipose tissue (eWAT) a non-statistically significant reduction (46.3 ± 20.1%; *p* = 0.09) was observed compared with control mice. In aTCF7L2hom mice, expression was reduced by 77.3 ± 11.2% and 57.8 ± 20.2%, respectively in eWAT (*p* = 0.008) and iWAT (*p* = 0.003) compared with control mice. Conversely, no changes in *Tcf7l2* expression were apparent in liver or pancreatic islets from aTCF7L2het and aTCF7L2hom mice compared with controls (Fig. 1a). Correspondingly, expression of the two TCF7L2 protein isoforms (79 kDa and 58 kDa) in eWAT was significantly reduced by 77 ± 17% and 80 ± 14% in aTCF7L2hom mice, while TCF7L2 protein levels were not different between aTCF7L2het and controls (Fig. 1b, c). Body weight in male (Fig. 1d) and female (Fig. 1e) aTCF7L2het and aTCF7L2hom mice was similar to that of control mice maintained on a normal chow diet (NC). No apparent changes in adipocyte morphology were observed in male mice maintained under NC (Fig. 1f). Likewise, adipose-selective *Tcf7l2* deletion did not affect fat or lean mass in 8-week-old male or female mice, as assessed by echoMRI (Fig. 1g, i, k, m). However, in older male aTCF7L2het mice, fat mass was increased (1.4-fold), and lean mass was decreased (0.9-fold; Fig. 1h, j) vs that in male control mice. Female mice showed no difference in body fat composition between the three groups (Fig. 1l and n).

### Adipocyte-selective *Tcf7l2* deletion leads to impaired glucose tolerance, with no effect on insulin sensitivity

**Fig. 2:**
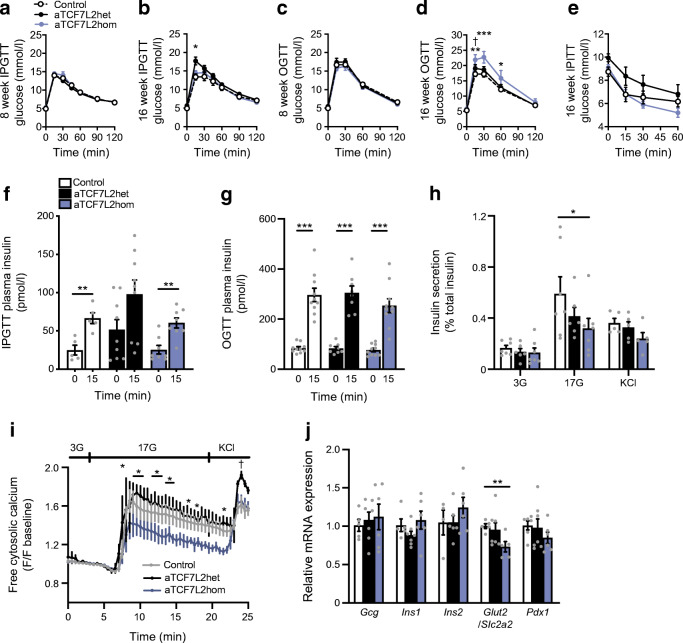
Effects of adipocyte-selective *Tcf7l2* deletion on glucose tolerance and beta cell function in male mice maintained on an NC diet. (**a**) IPGTT in 8-week-old male mice (*n* = 10 control mice, *n* = 10 aTCF7L2het mice, *n* = 9 aTCF7L2hom mice) maintained on an NC, and (**b**) in 16-week-old male mice (*n* = 10 control mice, *n* = 10 aTCF7L2het mice, *n* = 9 aTCF7L2hom mice). *p < 0.05 aTCF7L2het vs control group by two-way ANOVA with Tukey’s post hoc test. (**c**) OGTT in 8-week-old male mice (*n* = 10 control mice, *n* = 6 aTCF7L2het mice, *n* = 6 aTCF7L2hom mice), and (**d**) 16-week-old male mice (*n* = 10 control mice, *n* = 6 aTCF7L2het mice, *n* = 6 aTCF7L2hom mice). ***p < 0.001, **p < 0.01, *p < 0.05 aTCF7L2hom vs control and †*p* < 0.05 aTCF7L2hom vs aTCF7L2het by two-way ANOVA with Tukey’s post hoc test. (**e**) IPITT in 16-week-old mice (*n* = 10 control mice, *n* = 10 aTCF7L2het mice, *n* = 10 aTCF7L2hom mice). (**f**) Plasma insulin levels after i.p. injection of glucose (3 g/kg) in 16-week-old male mice (*n* = 5 control mice, *n* = 9 aTCF7L2het mice, *n* = 8 aTCF7L2hom mice). **p < 0.01 15 min vs 0 min condition by unpaired Student’s *t* test. (**g**) Insulin plasma levels after oral administration of glucose (3 g/kg) in 16-week-old male mice (*n* = 10 control mice, *n* = 7 aTCF7L2het mice, *n* = 9 aTCF7L2hom mice). ***p < 0.001 15 min vs 0 min condition by unpaired Student’s *t* test. (**h**) Insulin secretion by isolated islets from 17-week-old male mice (*n* = 5–7 mice/genotype). *p < 0.05 aTCF7L2hom vs control by two-way ANOVA with Tukey’s post hoc test. (**i**) Measurement of dynamic changes in intracellular calcium concentrations in isolated islets from 17-week-old male mice in response to perfusion of glucose (3 mmol/l, 3G; 17 mmol/l, 17G) and KCl (30 mmol/l) and represented as fold change of fluorescence intensity (F) compared with basal state at low glucose (*n* = 3 mice/genotype). *p < 0.05 aTCF7L2het vs aTCF7L2hom, †*p* < 0.05 aTCF7L2het vs control by two-way ANOVA with Tukey’s post hoc test. (**j**) mRNA expression profiling by quantitative RT-PCR of key pancreatic islet markers in isolated islets from 17-week-old male mice; each dot represents data from one mouse. **p < 0.01 aTCF7L2hom vs control by two-way ANOVA with Tukey’s post hoc test. Data are shown as mean ± SEM

**Fig. 3:**
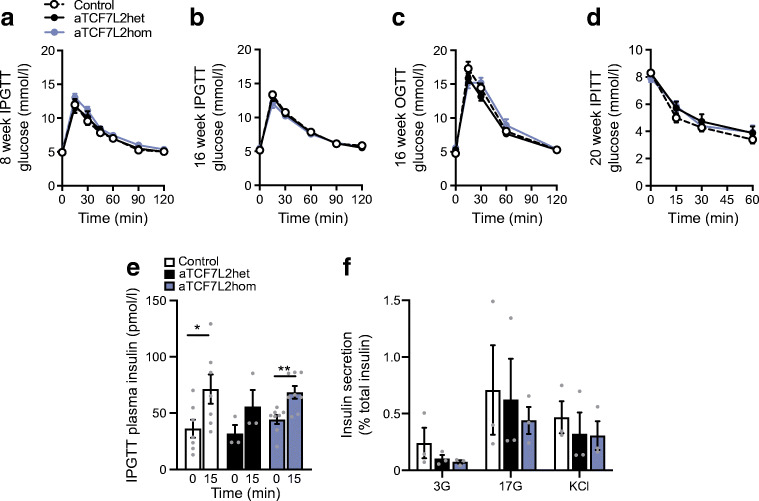
Effects of adipocyte-selective *Tcf7l2* deletion on glucose tolerance and beta cell function in female mice maintained on an NC. (**a**) IPGTT in 8-week-old female mice maintained on an NC (*n* = 10 control mice, *n* = 10 aTCF7L2het mice, *n* = 8 aTCF7L2hom mice) and (**b**) in 16-week-old female mice (*n* = 10 control mice, *n* = 10 aTCF7L2het mice, *n* = 8 aTCF7L2hom mice). (**c**) OGTT in 16-week-old female mice (*n* = 6 control mice, *n* = 9 aTCF7L2het mice, *n* = 4 aTCF7L2hom mice). (**d**) IPITT in 20-week-old female mice (*n* = 10 control mice, *n* = 7 aTCF7L2het mice, *n* = 5 aTCF7L2hom mice). (**e**) Insulin plasma levels after glucose injection (3 g/kg) in 20-week-old female mice on an NC (*n* = 7 control mice, *n* = 3 aTCF7L2het mice, *n* = 8 aTCF7L2hom mice); *p < 0.05, **p < 0.01 15 min vs 0 min condition by unpaired Student’s *t* test. (**f**) Insulin secretion on isolated islets from 20-week-old female mice during static incubation with 3 mmol/l glucose (3G), 17 mmol/l glucose (17G) and 30 mmol/l KCl, (*n* = 3 mice/genotype). Data are shown as mean ± SEM

Next, we explored the effects of adipocyte-selective *Tcf7l2* ablation on whole body glucose handling. Glucose challenge was performed in male and female mice at 8 and 16 weeks of age. At 8 weeks, blood glucose levels after i.p. injection of glucose were similar in aTCF7L2het and aTCF7L2hom mice and sex-matched littermate controls (Fig. 2a, Fig. 3a). At 16 weeks, glucose tolerance was impaired in male aTCF7L2het mice vs controls (17.7 ± 1.0 mmol/lvs 13.4 ± 0.8 mmol/l at 15 min after i.p. injection of glucose, *p* = 0.01; Fig. 2b). Eight-week-old aTCF7L2het and aTCF7L2hom mice had similar oral glucose tolerance to control mice (Fig. 2c). However, oral glucose tolerance was impaired in 16-week-old aTCF7L2hom mice compared with aTCF7L2het and control mice (*p* = 0.006 at 15 min, Fig. 2d). There were no differences in glucose tolerance between the three groups of female mice, regardless of age (Fig. 3a–c). For each sex, body insulin sensitivity was unaffected across the genotypes (Fig. 2e, Fig. 3d).

### Adipocyte-selective *Tcf7l2* deletion leads to defective pancreatic beta cell function

To assess whether impaired i.p. and oral glucose challenge in 16-week-old male aTCF7L2het and aTCF7L2hom mice, respectively (Fig. 2b and d), was due to defective insulin secretion, beta cell secretory capacity was measured. Plasma insulin levels were similar after i.p. glucose injection (Fig. 2f) or after oral glucose administration (Fig. 2g) in aTCF7L2het and aTCF7L2hom mice vs controls. Insulin secretion following stimulations with 17 mmol/l glucose was impaired in islets isolated from aTCF7L2hom mice vs that in islets from controls (0.32 ± 0.08% of vs 0.59 ± 0.13%, respectively, corresponding to a 0.54 ± 0.13-fold decrease; Fig. 2h), while responses to KCl (30 mmol/l) were not different between islets from aTCF7L2hom and those from control mice (Fig. 2h). Insulin secretion in aTCF7L2het islets showed no statistically significant differences in response to high glucose (*p* = 0.1) or KCl (*p* = 0.8) vs that in control islets.

To explore the origins of the insulin secretory defects observed in isolated islets (Fig. 2h), we measured changes in cytosolic free calcium (Ca^2+^) of isolated islets in response to incubation with varying concentrations of glucose (3–17 mmol/l) or KCl (Fig. 2i). Islets from aTCF7L2hom male mice showed a diminished glucose-induced rise in cytosolic Ca^2+^ compared with aTCF7L2het animals (*p* = 0.01 at *t* = 7.3 min), while no statistical differences in Ca^2+^ dynamics were observed compared with control mice (Fig. 2i). Islets from aTCF7L2het male mice showed an elevated Ca^2+^ response to KCl compared with controls (*p* = 0.01; Fig. 2i).

To examine the potential effects of adipose-selective *Tcf7l2* ablation on beta cell identity we measured the expression of signature genes associated with this and other islet cell types. No differences were observed in the expression of the insulin (*Ins1, Ins2*), or glucagon (*Gcg*) genes in islets across genotypes. However, a significant reduction in the expression of *Glut2* (also known as *Slc2a2*), usually confined to beta cells, was observed in aTCF7L2hom mice in comparison with controls (0.73 ± 0.06 in aTCF7L2hom vs 1.00 ± 0.03 in controls, *p* = 0.007; Fig. 2j). In vivo and in vitro insulin secretion was unchanged in female aTCF7L2het and aTCF7L2hom mice vs controls (Fig. 3e, f).

### Adipocyte TCF7L2 expression affects plasma levels of incretins and circulating NEFA

**Fig. 4:**
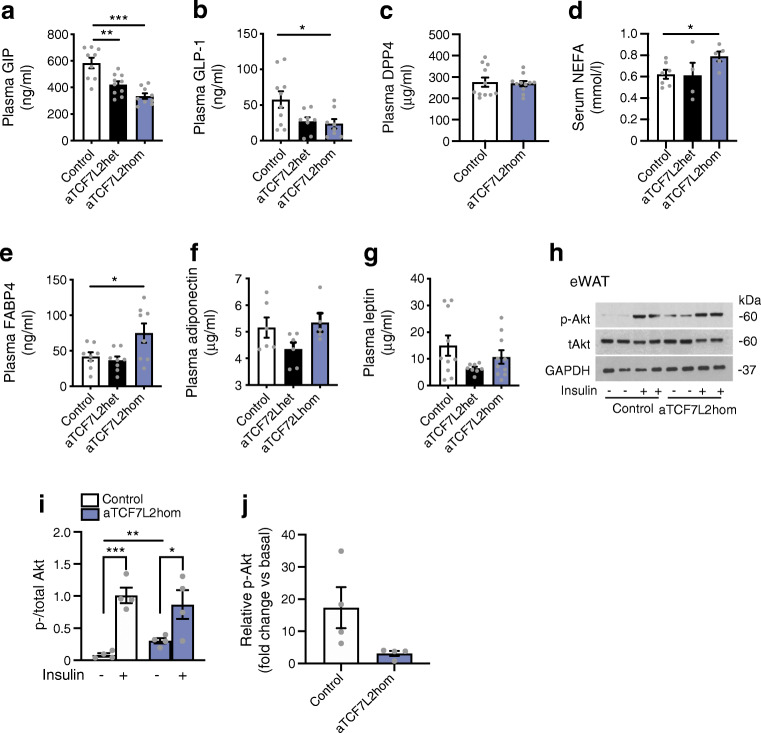
NEFA and incretin levels depend on adipocyte *Tcf7l2* expression. (**a**) GIP plasma levels in the fed state. ***p < 0.001 aTCF7L2homo vs control and **p < 0.01 aTCF7L2het vs control by one-way ANOVA with Tukey’s post hoc test. (**b**) GLP-1 plasma levels in the fed state. *p < 0.05 aTCF7L2hom vs control by one-way ANOVA with Tukey’s post hoc test. (**c**) DPP4 plasma levels. (**d**) NEFA serum levels. *p < 0.05 aTCF7L2hom vs control by one-way ANOVA with Tukey’s post hoc test. (**e**) FABP4 plasma levels. *p < 0.05 aTCF7L2hom vs control by one-way ANOVA with Tukey’s post hoc test. (**f**) Adiponectin plasma levels. (**g**) Leptin plasma levels. (**h**) Representative western blot of phosphorylated and total Akt from eWAT homogenates harvested from male mice 10 min after i.p. injection of sterile PBS (− insulin) or 1 U/kg of insulin (+ insulin) (*n* = 4 mice/genotype). (**i**) Densitometry analysis of *n* = 4 mice per genotype ***p < 0.001 insulin (+) vs insulin (−) condition in control by unpaired Student’s *t* test, **p < 0.01 insulin (−) in aTCF7L2hom vs insulin (−) control by unpaired Student’s *t* test, *p < 0.05 insulin (+) vs insulin (−) condition in aTCF7L2hom by unpaired Student’s *t* test. (**j**) Fold change of phosphorylated Akt expression after insulin stimulation over basal (− insulin). *p* = 0.07 aTCF7L2hom vs control by unpaired Student’s *t* test. Data shown as mean ± SEM

To investigate the causes of impaired oral glucose tolerance in male aTCF7L2hom mice, we measured the circulating levels of other factors known to be involved in the regulation of blood glucose in 16-week-old animals. Circulating GIP levels in randomly fed (i.e. had free access to food) male aTCF7L2het and aTCF7L2hom mice (Fig. 4a) were significantly decreased compared with those in age- and sex-matched littermate control mice (421.8 ± 24.6 ng/ml [*p* = 0.002] and 335.1 ± 21.4 ng/ml [*p* < 0.0001], respectively, vs 583.8 ± 39.5 ng/ml in controls, corresponding to a 0.4 ± 0.1-fold decrease). Likewise, circulating GLP-1 levels in randomly fed male aTCF7L2hom mice (Fig. 4b) were decreased compared with controls (23.7 ± 6.8 ng/ml vs 57.6 ± 11.6 ng/ml, *p* = 0.04, corresponding to a 0.6 ± 0.1-fold decrease). In aTCF7L2het mice, changes in GLP-1 levels did not reach statistical significance (27.1 ± 5.8 ng/ml, vs 57.6 ± 11.6 ng/ml, *p* = 0.06). Plasma DPP4 levels in male aTCF7L2hom mice were not different than those in controls (Fig. 4c).

Suggesting a role for adipose tissue TCF7L2 as a regulator of fatty acid release from these cells, plasma levels of circulating NEFA and the lipid carrier FABP4 were increased in aTCF7L2hom mice compared with age- and sex-matched littermate controls (NEFA: 0.79 ± 0.04 vs 0.62 ± 0.04 mmol/l, respectively, *p* = 0.03, corresponding to a 1.3 ± 0.1-fold increase; FABP4: 75.0 ± 13.5 ng/ml vs 42.1 ± 6.0 ng/ml, respectively, *p* = 0.05, corresponding to a 1.8 ± 0.3-fold increase; Fig. 4d, e). Plasma levels of adiponectin, leptin, resistin and PAI-1 in the fed state were not different in aTCF7L2het or aTCF7L2hom mice compared with those in littermate control mice (Fig. 4f, g; ESM Fig.1a, b).

We next explored whether the above effects of *Tcf7l2* deletion may reflect altered insulin signalling in adipocytes. Akt (also known as protein kinase B) Ser473 phosphorylation was elevated under basal conditions prior to insulin stimulation in aTCF7L2hom male mice (Fig. 4h, i). The difference in Akt phosphorylation after insulin stimulation (i.e. fold change above basal) did not reach statistical significance in adipocytes from aTCF7L2hom mice vs those from littermate controls (*p* = 0.07; Fig. 4j).

### Exposure to an HFD reduced insulin secretion in aTCF7L2hom mice

**Fig. 5:**
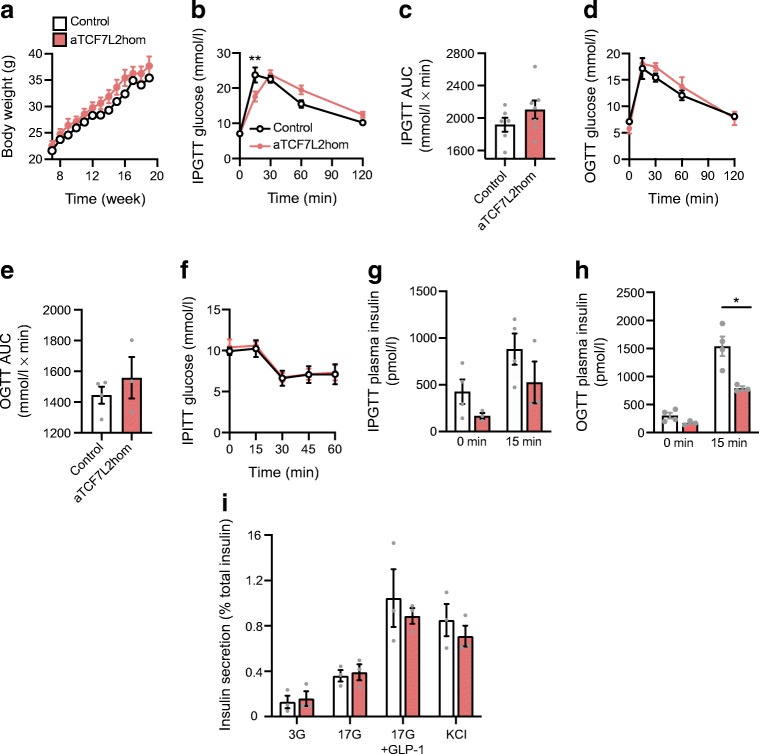
Effects of HFD on adipocyte-selective deletion of *Tcf7l2*. (**a**) Body weight in male mice during HFD feeding (*n* = 6 mice/genotype). (**b**) IPGTT in male mice after 9 weeks of HFD (*n* = 6 mice/genotype). **p = 0.003 aTCF7L2hom vs control by two-way ANOVA followed by Bonferroni post hoc test. (**c**) AUC corresponding to (**b**). (**d**) OGTT in male mice after 12 weeks of HFD (*n* = 4 control mice, *n* = 3 aTCF7L2hom mice). (**e**) AUC corresponding to (**d**). (**f**) IPITT in male mice after 12 weeks of HFD (*n* = 3 mice/genotype). (**g**) Insulin plasma levels after i.p. injection of glucose (2 g/kg) in male mice following 12 weeks of HFD (*n* = 4 control mice, *n* = 3 aTCF7L2hom mice). (**h**) Insulin plasma levels after oral administration of glucose (2 g/kg) in male mice following 12 weeks of HFD (*n* = 4 control mice, *n* = 3 aTCF7L2hom mice). *p < 0.05 aTCF7L2hom vs control at 15 min condition by unpaired Student’s *t* test. (**i**) Insulin secretion on isolated islets from male mice after 12 weeks of HFD during static incubation of glucose (3 mmol/l, 3G; 17 mmol/l, 17G), a combination of 17 mmol/l glucose and 20 nmol/l GLP-1 (17G+GLP-1) and KCl (30 mmol/l), (*n* = 3 mice/genotype). Data shown as mean ± SEM

To determine whether the absence of TCF7L2 in adipocytes may influence the response of glucose homeostasis to a metabolic stress, we maintained male aTCF7L2hom or control mice on an HFD for up to 12 weeks. No significant differences in changes in body weight over time were observed between aTCF7L2hom mice and littermate controls (Fig. 5a). After 9 weeks’ exposure to an HFD, a delayed blood glucose peak was observed in aTCF7L2hom compared with controls in response to i.p. glucose injection (Fig. 5b). No significant differences were observed between the two groups of mice during OGTT (Fig. 5d, e) or in insulin sensitivity (Fig. 5f) after exposure to an HFD for 12 weeks. Insulin secretion was impaired (at 15 min, 526.2 ± 223.1 in aTCF7L2hom vs 881.9 ± 166.9 pmol/l in control, *p* = 0.02, corresponding to a 0.5 ± 0.1-fold decrease; Fig. 5h) during in vivo oral glucose challenge in aTCF7L2hom mice compared with littermate control mice, while no differences in plasma insulin content were observed following i.p. glucose injection (Fig. 5g). Ex vivo insulin release in response to glucose (17 mmol/l), GLP-1 (20 nmol/l) and KCl (30 mmol/l) was found to be no different between islets isolated from aTCF7L2hom mice and control mice following 12 weeks of an HFD (Fig. 5i).

## Discussion

The overall aim of the present study was to explore the possibility that changes in *Tcf7l2* expression in the adipocyte may affect whole body glucose homeostasis and, if so, to explore the mechanisms involved. While our studies mimic alterations in *TCF7L2* expression that may occur in human adipose tissue as a function of rs7903146 genotype (see below), the extent of these changes in the mouse adipocyte are likely to be of much greater magnitude. We show that forced changes in *Tcf7l2* expression in murine adipose tissue leads to alterations not only in adipocyte function but also to the function of other tissues involved in the regulation of energy homeostasis in a sex- and age-dependent manner. Of note, the severity of the phenotypes was broadly dependent on the extent of *Tcf7l2* perturbation.

Wnt signalling involves an association between β-catenin and a member of the TCF family, such as TCF7L2, TCF7, TCF7L1 or lymphoid enhancer-binding factor-1 (LEF-1) [1]. The availability of free β-catenin able to enter the nucleus and bind TCF7L2 is crucial to activate downstream target genes. However, the regulation of TCF7L2 content is also important [20]. Previous studies have suggested that variation in *Tcf7l2* expression alters glucose metabolism and induces a type 2 diabetes phenotype [21], and that high-fat feeding modulates *Tcf7l2* expression in pancreatic islets, hepatocytes and adipocytes [22–24]. We show here that the extent *Tcf7l2* ablation impacts metabolic outcome. Thus, deletion of a single *Tcf7l2* allele led to impaired tolerance of glucose administrated intraperitoneally, and generated distinct features of obesity-induced glucose intolerance, while biallelic *Tcf7l2* deletion impacted on the oral glucose tolerance, it might exert an effect through endocrine signalling molecules such as the incretins. We note that the impact of the deletion of a single *Tcf7l2* allele on oral glucose and GLP-1 levels in randomly fed mice may not have reached significance due to the low number of mice analysed. A more thorough examination in the differences in phenotype could not be conducted in the present study because of disruptions to the breeding and experimental programme resulting from the coronavirus disease-2019 (COVID-19) pandemic. These questions merit further investigation at a future date.

Wnt and its effectors (β-catenin and TCF7L2) are critical for adipogenesis [14–16]. However, the persistence of this signalling module into adulthood indicates that it is also important in the mature adipocyte. In the present study, we found that young mice lacking TCF7L2 in the adipocyte displayed no alteration of glucose tolerance or body composition, while defects appeared with age in male mice (Fig. 1g, h, Fig. 2a, b). Interestingly, female mice were largely unaffected by the loss of TCF7L2 from the adipocyte (Fig. 3), consistent with a role for female hormones in the maintenance of glucose homeostasis and differences in fat distribution between male and female mice (Figs 1, 2 and 3). Correspondingly, Tian et al [25] have previously revealed crosstalk between Wnt signalling and female hormones through TCF7L2. We therefore conducted our studies on the effects of HFD exposure (see below) in male mice only.

Impaired adipocyte function has previously been shown to impact beta cell function by various mechanisms, including the release of adipokines (reviewed in [26]). Male mice lacking both *Tcf7l2* alleles selectively in adipocytes and maintained on an NC diet displayed an impaired response to oral glucose challenge (Fig. 2d) but normal tolerance to i.p. injection of the sugar (Fig. 2b). This suggests an abnormal incretin effect, defined as the postprandial insulin response provoked by incretin hormones such as GLP-1 and GIP. However, insulin release in response to oral glucose was maintained (Fig. 2g) with lowered circulating levels of GIP and GLP-1 (Fig. 4a, b), while glucose-stimulated insulin secretion ex vivo from isolated islets was impaired (Fig. 2h). Our data therefore suggest that a mechanism exists to maintain insulin release in vivo after deletion of *Tcf7l2* from adipocytes when the incretin effect is compromised. One possible explanation for this difference between in vivo and ex vivo glucose-stimulated insulin secretion is that elevated fatty levels compensate in part for the lowered levels of circulating incretins, acting to amplify insulin release through the action of fatty acid receptors. NEFA, and specifically long-chain fatty acids, potentiate glucose-stimulated insulin secretion [27, 28]. Moreover, a direct insulinotropic action of FABP4—a cytosolic lipid chaperone expressed and secreted by white and brown adipocytes whose levels were increased in aTCF7L2hom mice, as described below—may act directly on pancreatic beta cells, as demonstrated in previous studies showing that recombinant FABP4 administration enhanced glucose-stimulated insulin secretion in vitro and in vivo [29, 30]. This is consistent with recently published data indicating that an elevated FABP4 level is associated with the incidence of type 2 diabetes in humans [31, 32].

How might depletion of *Tcf7l2* from adipocytes lead to a decrease in the circulating levels of GLP-1 and GIP? Decreased plasma incretin content in aTCF7L2hom mice is unlikely to be due to an increase in the rate of degradation of these hormones in the bloodstream, as no change was found in circulating DPP4 in aTCF7L2hom mice (Fig. 4c). Providing potential candidates as regulators of the corresponding enteroendocrine cells, we observed elevated circulating NEFA and FABP4 levels (Fig. 4d, e), after TCF7L2 deletion from the adipocyte. However, *Fabp4* expression at the mRNA level was not altered in aTCF7L2hom mice, suggesting regulation at a later stage in biosynthesis, or conceivably an alternative source of the adipokine, such as the liver. In addition, our measurements of GLP-1 and GIP were performed in randomly fed mice. Future experiments might include a more in-depth analysis of the release of the incretins in response to an oral glucose load and an assessment of whether incretin release is affected by adipose-selective *Tcf7l2* deletion in mice that have been maintained on an HFD. Measurements of food intake and energy expenditure would also be of merit.

Wnt and TCF7L2 are regulators of serum and hepatic triacylglycerol content [25], consistent with elevated plasma triacylglycerol associated with risk rs7903146 alleles [33]. Martchenko and colleagues [34] have recently reported fatty acid-induced lowering of circadian release of GLP-1 from L cells as a result of decreased *Bmal1* expression. Similar findings on the inhibition by fatty acids of GLP-1 secretion have been reported by others [35, 36]. Activation of fatty acid receptors (FFARs) with the FFAR1/GPR40 agonist TAK-875 [37] or with short-chain fatty acids (FFAR2/GPR43) [38] acutely increases GLP-1 secretion from L cells. This suggests a balance between positive shorter term effects and more chronic ‘lipotoxic’ effects of NEFA may govern overall incretin production, with the latter predominating after *Tcf7l2* deletion in adipocytes.

To explore further the direct role of TCF7L2 in lipid metabolism, future studies will be necessary to assess lipolysis in adipocytes lacking TCF7L2 and peripheral insulin action. Finally, we note that selective loss of TCF7L2 from mouse beta cells lowers GLP-1 receptor expression [3]. This is in line with data showing that carriers of the risk allele of rs7903146 exhibit incretin resistance [39, 40].

Our data would appear partly to contradict a previous study wherein post-glucose challenge GLP-1 levels were not different between human carriers of the risk vs non-risk allele of rs7903146 [40]. However, the knockout strategy that we employed in the mouse results in a much more drastic change in *Tcf7l2* gene expression than that observed in humans bearing the risk allele of rs7903146. We note that we measured plasma GIP and GLP-1 levels in randomly fed mice; measurement of GIP and GLP-1 responses to a glucose bolus in aTCF7L2 knockout and control mice may provide a better indication as to whether secretion from the relevant enteroendocrine cells, rather than subsequent metabolism of the peptides, is affected.

In vivo glucose-stimulated insulin secretion in aTCF7L2hom male mice was impaired by exposure to an HFD (Fig. 5h), a finding reminiscent of the impact of this regimen on TCF7L2 deletion-induced impairments in insulin secretion [2, 3, 41]. Surprisingly, however, high-fat feeding had only minor effects on the impact on glucose tolerance. Future studies will need to assess the effects of an HFD on incretin and insulin secretion in beta cells, and to characterise the impact of an HFD on aTCF7L2het mice, as explorations were suspended during the COVID-19 pandemic. The size of the cohorts should be increased in future studies to determine the impact of an HFD. A further limitation of the present study is the lack of examination of a potential role for incretins in the impaired glucose tolerance observed in mice lacking TCF7L2 in adipocytes.

We would stress that there are several differences between our results and previous studies on TCF7L2 function in mouse adipocytes. When examining the effects of conditional deletion *Tcf7l2* in these cells, Chen et al [16] observed impairments in glucose tolerance after i.p. injection of glucose in 3-month-old male and female mice under standard diet associated with hepatic insulin resistance. In contrast, we observed no changes in glucose tolerance in NC-fed aTCF7L2 knockout female mice. Geoghegan et al [13] also generated a conditional knockout of *Tcf7l2* in the adipocyte, reporting that knockout animals maintained on regular chow displayed no change in i.p. glucose tolerance, while exaggerated insulin resistance and impaired glucose tolerance were apparent after high-fat feeding [13]. The authors demonstrated a role for TCF7L2 in regulating lipogenic and lipolytic gene expression, finding impaired lipolysis in response to fasting. We note that slightly different genetic strategies were used to reduce TCF7L2 expression in these studies. Whereas we deleted exon 1 of *Tcf7l2*, Chen et al targeted exon 11 and Geoghegan et al targeted exon 5 [13, 16]. Genetic background, housing and experimental conditions may also have contributed to the differences observed.

Might changes in TCF7L2 expression in adipose tissue contribute to the effects of type 2 diabetes-associated variants in humans? Although rs7903146 variants in *TCF7L2* are not associated with changes in overall *TCF7L2* levels (i.e. the total of all isoforms), there are conflicting expression quantitative trait loci (eQTL) data regarding the association of rs7903146 with the expression of specific *TCF7L2* splice variants in subcutaneous fat [42–44]. These differences may reflect sample size and disease heterogeneity [45, 46]. Nevertheless, Mahajan and colleagues [47] recently identified genomic variants in the *TCF7L2* gene in linkage disequilibrium with rs7903146 which map to adipose and liver enhancers, and may therefore influence *TCF7L2* expression in these tissues. In addition, surgery-induced weight loss [48], plasma triacylglycerol and NEFA levels [49] have been shown to influence alternative splicing of *TCF7L2* in adipose tissue, with other evidence indicating that acute intake of fat leads to reduced expression of *TCF7L2* in human adipocytes [24]. Overall, these data suggest that changes in *TCF7L2* expression may be linked to adaptation to changes in fuel intake. While inspection of data in the GTEX database [50] does not reveal any genotype-driven alteration in subcutaneous or breast adipose tissue *TCF7L2* expression with rs7903146 genotype, studies on larger numbers of individuals may now be warranted.

### Conclusion

We demonstrate here a critical role for adipocyte TCF7L2 in systemic glucose homeostasis in mice. Thus, we provide unexpected insights into the action of TCF7L2, revealing a novel mechanism through which changes in the expression of this gene in adipocytes has sex-specific effects on glucose homeostasis, and may impact both insulin and incretin secretion. Although our data do not directly model the effects of rs7903146 on type 2 diabetes risk, in revealing a new level of complexity in diabetes-related gene action at the systems level, our findings may help in the development of novel personalised therapies.

## Electronic supplementary material

ESM (PDF 224 kb)

## Data Availability

Data presented in this manuscript are available upon request from the corresponding authors.

## Abbreviations

aTCF7L2het: (Mice with) genetic ablation of a single *Tcf7l2* gene in mature adipocytes
aTCF7L2hom: (Mice with) genetic ablation of both *Tcf7l2* genes in mature adipocytes
COVID-19: Coronavirus disease-2019
DPP4: Dipeptidyl peptidase 4
eWAT: Epididymal white adipose tissue
FABP4: Fatty acid binding protein 4
GIP: Glucose-dependent insulinotropic polypeptide
GLP-1: Glucagon-like peptide-1
HFD: High-fat diet
HTRF: Homogeneous time resolved fluorescence
iWAT: Inguinal white adipose tissue
NC: Normal chow diet
PAI-1: Plasminogen activator inhibitor-1
TCF7L2: Transcription factor 7-like 2
