# Supplementary material for: Adipocyte-specific deletion of Tcf7l2 induces dysregulated lipid metabolism and impairs glucose tolerance in mice
Source: Diabetologia. 2020 Oct 17;64(1):129–41. doi: 10.1007/s00125-020-05292-4 (PMC7567653; doi:10.1007/s00125-020-05292-4)

Animals

To achieve tissue-selective ablation of *Tcf7l2* alleles, we crossed mice in which exon 1 (encoding the beta-catenin-binding domain) was flanked by *LoxP* sites [1] to mice expressing *Cre* recombinase under the control of the adiponectin promoter [2] (a kind gift from D. Withers, Imperial College London) to produce deletion of a single (aTCF7L2het) or two *Tcf7l2* alleles (aTCF7L2hom). Littermates used as controls did not express *Cre* recombinase but were homozygous or heterozygous for the floxed *Tcf7l2* allele. Adiponectin-*Cre* [2] mice, or animals with *Tcf7l2* gene flanked by LoxP sites (TCF7L2-floxed) [1], did not display metabolic phenotypes that deviated from wild-type littermate control mice, consequently we used *Tcf7l2*-floxed mice as controls in our test cohorts.

In vivo metabolic assays

Glucose tolerance was performed on 15 h-fasted mice after an oral gavage of glucose (2 g/kg of body weight) or intraperitoneal injection of glucose (1g/kg body weight). IPGTT and OGTT were performed at two stages (at 8-week-old and at 16-week-old) for each individual mouse. Insulin tolerance was performed after a 5 h-fast with an intraperitoneal injection of insulin (0.5 U/kg in females, 0.75 U/kg in males under chow diet, 1.5 U/kg in males under HFD). *In vivo* glucose-stimulated insulin secretion was assessed after oral or intraperitoneal administration of glucose and blood was collected at 0- and 15-minutes post-injection to assess plasma insulin levels using an ultra-sensitive mouse insulin ELISA kit (Crystal Chem, Netherlands) or using a Homogeneous Time Resolved Fluorescence (HTRF) insulin kit (Cisbio, France) in a PHERAstar reader (BMG Labtech, UK).

In vitro insulin secretion

Insulin secretion assays on isolated mouse islets were performed as previously described [3]. In brief, 10 size-matched islets per condition were incubated for 1h in Krebs-HEPES-bicarbonate (KHB) solution (130 mmol/l NaCl, 3.6 mmol/l KCl, 1.5 mmol/l $CaCl_2$, 0.5 mmol/l $MgSO_4$, 0.5 mmol/l KH_2PO_4, 2 mmol/l $NaHCO_3$, 10 mmol/l HEPES, and 0.1% BSA, pH 7.4) containing 3 mmol/l glucose. Subsequently, islets were incubated for 30 minutes in KHB solution with either 3 mmol/l-glucose, 17 mmol/l-glucose or 30 mmol/l-KCl. Secreted and total insulin were quantified using a HTRF insulin kit (Cisbio, France) in a PHERAstar reader (BMG Labtech, UK) following the manufacturer's guidelines.

Protein isolation and Western immunoblotting

To assess TCF7L2 expression, epididymal adipose tissues were collected and frozen in liquid nitrogen in a fed state. To assess insulin sensitivity, male mice were starved for four hours and following an intraperitoneal injection of insulin (1 IU/kg body weight) adipose tissues were collected and frozen in liquid nitrogen. Adipose tissue proteins were extracted in lysis buffer (150 mmol/l NaCl, 50 mmol/l

Tris-HCl pH 8.0, 1% NP-40) supplemented with protease inhibitors cocktail (Roche, Germany) and phosphatase inhibitors cocktail (Sigma-Aldrich, UK) and analysed by Western blotting using antibodies for TCF4/TCF7L2 (C48H11) (#2569, 1:500, Cell signalling, NEB, UK), phospho-AKT (#9271, 1:1000, Cell signalling, NEB, UK), total-AKT (#9272, 1:1000, Cell signalling, NEB, UK), GAPDH (#2118, 1:10000, Cell signalling, NEB, UK), alpha-tubulin (T5168, 1:10 000, Sigma-Aldrich, UK). Fiji software was used for densitometry quantification.

Pancreatic islet isolation

Islets were isolated by digestion with collagenase as described [3]. In brief, pancreata were inflated with a solution of collagenase from *clostridium histolyticum* (1 mg/ml; Nordmark, Germany) and placed in a water bath at 37 ºC for 12 min. Islets were washed and purified on a Histopaque gradient (Sigma-Aldrich, UK). Isolated islets were cultured for 24 h in RPMI 1640 containing 11.1 mmol/l glucose, 10% foetal bovine serum and L-glutamine (Sigma-Aldrich, UK) and allowed to recover overnight.

Intracellular free calcium imaging

Measurement of intracellular calcium dynamics was performed as previously described [4]. In brief, whole isolated islets were incubated with fura-8AM (Invitrogen, UK) [5] for 45 min at 37ºC in KHB containing 3 mmol/l glucose. Fluorescence imaging was performed using a Nipkow spinning disk head (Yokogawa CSU-10; Runcorn, UK), allowing rapid scanning of islet areas for prolonged periods of time with minimal phototoxicity. Volocity software (PerkinElmer Life Sciences, UK) provided interface while islets were kept at 37ºC and constantly perifused with KHB containing 3 mmol/l or 17 mmol/l glucose or 30 mmol/l KCl.

Analysis of circulating factors in plasma and serum

Catalogue numbers for ELISA, colorimetric and protein array kits: GLP-1, GIP, leptin, adiponectin and PAI-1 kit 17004350 Bio-Plex Pro Mouse Diabetes, Biorad, UK; FABP4 and resistin kit LXSAMSM-01/02/06 Mouse Magnetic Luminex Assay R&D Systems, UK; serum NEFA FA115, Randox, UK; DPP4 ELISA kit DY954, R&D Systems, UK.

Histology

Epididymal adipose tissue was harvested, fixed overnight in 10% (v/v) formalin and embedded in paraffin wax. Tissue slices (5 μm) were stained with H&E (Sigma-Aldrich, UK) for morphological analysis using a widefield Axiovert 200M microscope (Zeiss, Germany) in the Facility for Imaging by Light Microscopy (Imperial College London).

RNA isolation and quantitative PCR

RNA was isolated from epididymal and subcutaneous adipose tissue, liver and pancreatic islets with TRIzol following manufacturer's instructions (Invitrogen, UK). RNA purity and concentration were measured by spectrophotometry (Nanodrop, Thermo Scientific, UK). Only RNA with absorption ratios between 1.8-2.0 for 260/280 and 260/230nm were used. RNA integrity was checked on an agarose gel. RNA was reversed transcribed using High-Capacity cDNA Reverse Transcription kit (Applied Biosystems, UK). qPCR was performed with Fast SYBR green master mix (Applied Biosystems, UK). The comparative Ct method ($2^{-\Delta\Delta CT}$) was used to calculate relative gene expression levels using *Gapdh*, *βactin* or *Ppia* as an internal control. The primers sequences are listed in Supplemental table S1.

Statistical analysis

GraphPad Prism 8.4 was used for statistical analysis (GraphPad Software, USA). Significance was evaluated by unpaired Student t -tests and one- or two-way ANOVA, with Tukey's multiple comparison test. A p value of <0.05 was considered statistically significant. Data are shown as mean ± SEM.

[1] da Silva Xavier G, Mondragon A, Sun G, et al. (2012) Abnormal glucose tolerance and insulin secretion in pancreas-specific Tcf7l2-null mice. Diabetologia 55(10): 2667-2676. 10.1007/s00125-012-2600-7

[2] Eguchi J, Wang X, Yu S, et al. (2011) Transcriptional control of adipose lipid handling by IRF4. Cell Metab 13(3): 249-259. 10.1016/j.cmet.2011.02.005

[3] Ravier MA, Rutter GA (2010) Isolation and culture of mouse pancreatic islets for ex vivo imaging studies with trappable or recombinant fluorescent probes. Methods Mol Biol 633: 171-184. 10.1007/978-1-59745-019-5_12

[4] Mitchell RK, Nguyen-Tu MS, Chabosseau P, et al. (2017) The transcription factor Pax6 is required for pancreatic beta cell identity, glucose-regulated ATP synthesis, and Ca(2+) dynamics in adult mice. J Biol Chem 292(21): 8892-8906. 10.1074/jbc.M117.784629

[5] Hodson DJ, Mitchell RK, Bellomo EA, et al. (2013) Lipotoxicity disrupts incretin-regulated human beta cell connectivity. J Clin Invest 123(10): 4182-4194. 10.1172/JCI68459

ESM Table 1: Primer sequences used for RT-qPCR analysis in this study.

Target	Reverse	Forward
Tcf7l2 exon 1	CTTGGCCGCTTCTTCCAA	CAAAACAGCTCCTCCGATTCC
Gcg	CCTTCAGCATGCCTCTCAAAT	CCAAGAGGAACCGGAACAAC
Ins1	AATGACCTGCTTGCTGATGGT	GCTGGTGGGCATCCAGTAA
Ins2	AGCTCCAGTTGTGCCACTTGT	CGTGGCTTCTTCTACACACCC
Pdx1	CCGCCAACTTCTCGTATTTCTC	CAAAGCTCACGCGTGGA
Glut2/Slc2a2	GCTTTGATCCTTCCAAGTTTGTC	TTACAGTCACACCAGCATACAC

ESM Fig.1: Circulating plasma levels of resistin (a) and PAI-1 (b) in male mice on chow diet. Blood was collected in the fed state. Each dot represents one mouse. Data shown as mean ± SEM.

ESM Fig.1: Plasma circulating of resistin (a) and PAI-1 (b) in male mice on chow diet, Each dot represents one mouse. Data shown as mean ± SEM

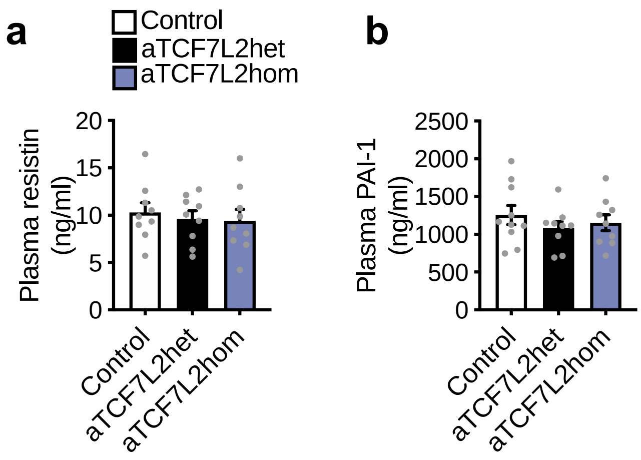

Supplement: Supplementary file 1 — (PDF 224 kb) [file 125_2020_5292_MOESM1_ESM.pdf]
